# Nitrogen supply and intercropping control of Fusarium wilt in faba bean depend on organic acids exuded from the roots

**DOI:** 10.1038/s41598-021-89109-3

**Published:** 2021-05-05

**Authors:** Jiaxing Lv, Jingxiu Xiao, Zengpeng Guo, Kun Dong, Yan Dong

**Affiliations:** 1 College of Resources and Environment, Yunnan Agricultural University, Kunming, 650201 China; 2 College of Animal Science and Technology, Yunnan Agricultural University, Kunming, 650201 China

**Keywords:** Parasitism, Biotic

## Abstract

Fusarium wilt in faba bean (*Vicia faba* L.) is caused by *Fusarium oxysporum* f. sp. *fabae* (FOF), which reduces the yield of crop. We used greenhouse, field and laboratory experiments to evaluate the role of organic acids in the occurrence of Fusarium wilt of faba bean to confirm the mechanism of rational application of nitrogen (N) and intercropping to alleviate Fusarium wilt. We investigated the response of organic acids exuded from the roots of faba bean to different N levels and cropping patterns (monocropping and intercropping with wheat). The results showed that the application of N and intercropping with wheat could control the Fusarium wilt of faba bean, which was closely related to the components and quantity of organic acids exuded from its roots. Among them, tartaric acid and malic acid are the most abundant and important, because they have a significant inhibitory effect on the growth and reproduction of FOF and substantially aid in the control of Fusarium wilt. The application of 90 kg ha^−1^ of N combined with wheat intercropping significantly controlled the Fusarium wilt and increased the grain yield of faba bean. Our results suggest that 90 kg ha^−1^ of N combined with intercropping is the most effective way to control Fusarium wilt and should be incorporated into agricultural management practices.

## Introduction

Faba bean (*Vicia faba* L.) is an important grain legume crop, particularly in China, Mediterranean countries and the Middle East^1^. The global acreage of faba bean is approximately 2.50 million ha in 2018 (FAOSTAT). China is the largest producer in the world, and, in 2018, the area of faba bean harvest reached 8.7 × 10^5^ ha, accounting for 34.5% of the global total (FAOSTAT), but these plants are primarily susceptible to Fusarium wilt disease during industrialized cultivation^2^. Fusarium wilt is harmful to faba bean production worldwide and limits the sustainable development of faba bean. *Fusarium oxysporum* f. sp. *fabae* (FOF) is the main pathogen of Fusarium wilt, it can survive in soil for many years without host, which brings many difficulties to control Fusarium wilt^3^. Domestic and foreign scholars have tried to use resistant varieties or chemical fungicides to control Fusarium wilt, but these methods are not environmentally friendly, effective, economical^4–7^. Scholars have found that increasing biodiversity is an effective measure to continuously control crop diseases, and intercropping is widely used as the most effective method to increase biodiversity^8–10^. In a survey, compared with monocropping, intercropping reduced disease severity caused by bacteria, fungi and viruses by more than 79%^11^.

Root exudates have been suggested to play a central role in influencing the interactions with neighboring plants and microbes^12^, and organic acids are among the most important components. Organic acids are incomplete oxidation products of photosynthetic assimilation products, which are either converted into carbohydrates or eventually oxidized to carbon dioxide and water. Their carbon skeleton can also be used to synthesize amino acids. The "intermediate" nature of the organic acids influences their diverse roles^13^. It is well known that organic acids are thought to be involved in many rhizosphere processes, including nutrient acquisition and metal detoxification, the alleviation of anaerobic stress in roots, mineral weathering and the attraction of beneficial bacteria^13^. For example, Momma et al. found that the secretion of organic acids by plants inhibited the growth of pathogens in the soil, including more than a dozen fungi such as *Fusarium oxysporum*^14^. Therefore, the changes in organic acids may provide an important clue to control Fusarium wilt of faba bean.

Inorganic nutrients are necessary in the process of crop growth and development, and fertilization is an effective measure to improve crop yield. Evidence shows that continuous cropping obstacle, particularly soil-borne diseases, can be controlled by nutritional regulation, since inorganic nutrients play an important role in the occurrence and development of many fungal and bacterial diseases^15^, and many inorganic nutrients can inhibit the occurrence of fungal diseases and the damage they cause^16^. The application of pure nitrogen (40 kg ha^−1^) reduced the number of *Fusarium* colonies in the rhizosphere of pigeon pea (*Cajanus cajan*) and the damage from Fusarium wilt^17^. Huang et al. concluded that urea combined with calcium superphosphate could reduce Fusarium wilt of watermelon^18^. Current research originates primarily from the observation of phenomena, and there are few studies on the mechanism of disease control. Studies have shown that N can change the root environment and affect plant resistance and sensitivity to disease by regulating plant metabolism during the process of plant–pathogen interactions^19^, and the increase of N fertilizer could cause changes in root exudates^20^. Therefore, we hypothesize that the application of nitrogen fertilizer can control Fusarium wilt of faba bean, and that it is more effective in intercropping systems, which may be closely related to the changes in organic acids. The objectives of this study were to (1) examine the responses of the organic acid exudates of faba bean roots to different N levels and cropping patterns (monocropping and intercropping), (2) evaluate the role of organic acids in the occurrence of Fusarium wilt of faba bean, and (3) provide theoretical guidance for the rational application of N fertilizer in a wheat/faba bean intercropping system.

## Results

### Effects of the application of N and intercropping on the organic acid exudates from faba bean roots from the greenhouse

**Figure 1:**
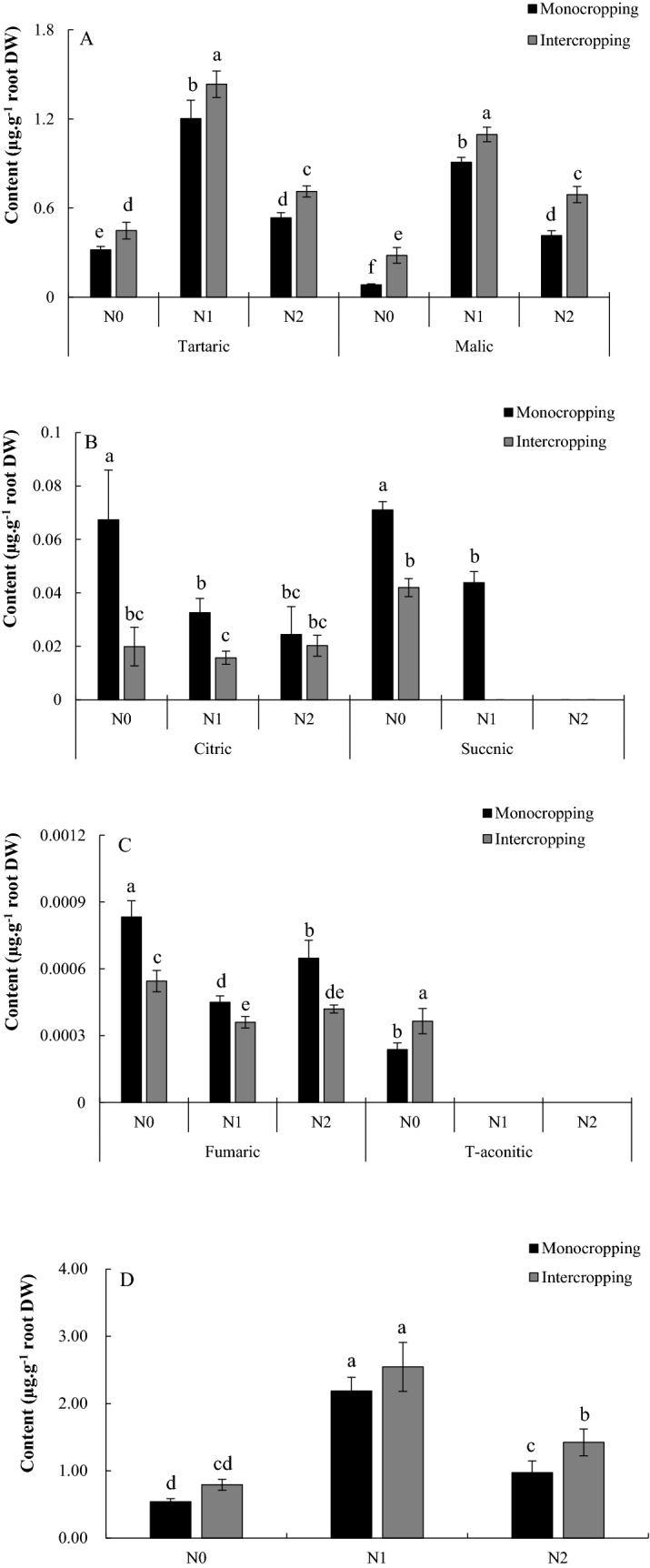
Quantity of organic acids in the root exudates of faba bean and total organic acids. Tartaric and Malic (**A**), Citric and Succnic (**B**), Fumaric and T-aconitic (**C**), Total organic acids (**D**). All the values are presented as the mean ± SE. The different letters on the mean values of the same organic acid indicate significant differences among the treatments (*P* < 0.05).

Six types of organic acids were detected in the root exudates of faba bean, including tartaric, malic, citric, succinic, fumaric and T-aconitic acids (Fig. 1). With the increase in application of N, the concentration of organic acids exuded from faba bean roots changed significantly. Compared with the N0 treatment, the amounts of tartaric acid and malic acid in the N1 and N2 treatments increased significantly, and those in the N1 treatment were the highest, which was the case for both monocropping and intercropping conditions (Fig. 1). In contrast, the amount of fumaric acid was the lowest in N1 treatment. The amount of citric acid decreased with the increase in application of N. Succinic acid was not detected in the N2 treatment, and T-aconitic acid was not detected in the N1 and N2 treatments. Compared with monocropping, intercropping significantly increased the amounts of tartaric acid and malic acid under the conditions of N0, N1 and N2, which were 40.74%, 19.18%, 33.26% and 236.4%, 20.56%, and 55.50% respectively. The amount of citric acid produced during intercropping decreased significantly under N0 and N1, but there was no significant difference under N2 compared with monocropping. Intercropping significantly increased the amount of T-aconitic acid under N0 conditions. Succinic acid was not detected under N1 intercropping conditions. In terms of total quantity, the application of N increased the exudation of organic acids from faba bean roots, and these organic acids reached their highest level under N1 conditions (Fig. 1D). Compared with monocropping, intercropping increased the exudation of organic acids, significant under N2 conditions (Fig. 1D).

### Effects of the organic acid exudates from faba bean roots on the growth of FOF

**Figure 2:**
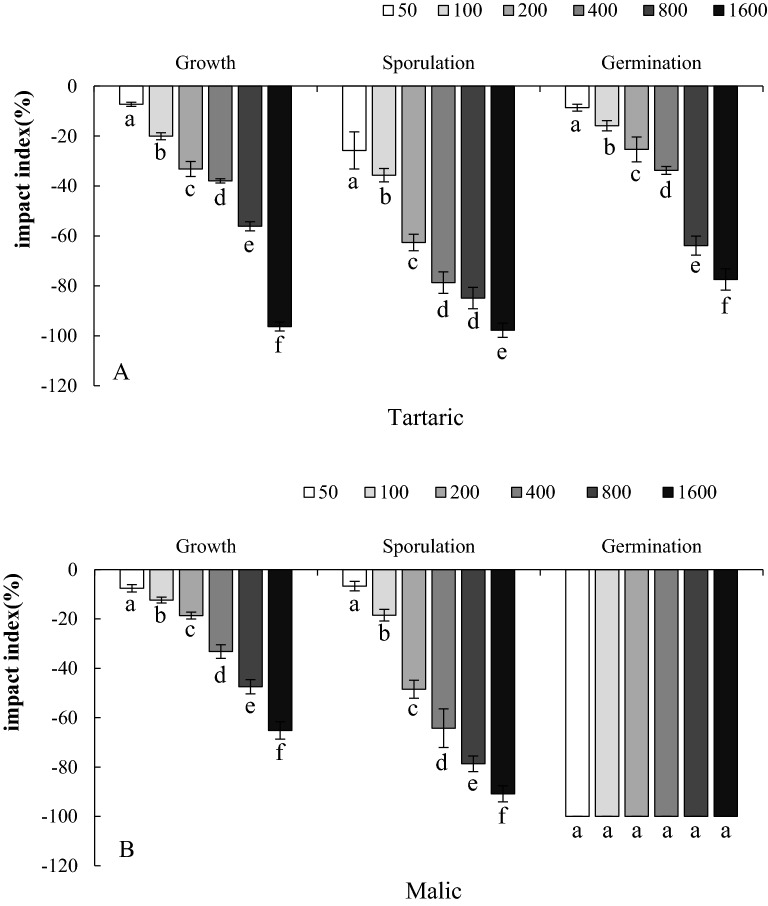
Effect of different concentrations of organic acids on the mycelial growth, sporulation and spore germination of FOF. Tartaric (A), Malic (B). All the values are presented as the mean ± SE. The different letters on the mean values of the same index indicate significant differences among the treatments (*P* < 0.05).

We chose tartaric acid and malic acid because they were present at the highest levels and added a wide range of concentrations to detect their effect on the growth of FOF. Tartaric acid inhibited the mycelial growth, sporulation and germination of spores of FOF at different concentrations, and the inhibition increased in parallel with the increase in concentration. Malic acid also exhibited a similar trend, and each concentration of malic acid inhibited the amount of germination of FOF spores by 100% (Fig. 2).

### Effects of the application of N and intercropping on the growth and yield of faba bean from the field

**Figure 3:**
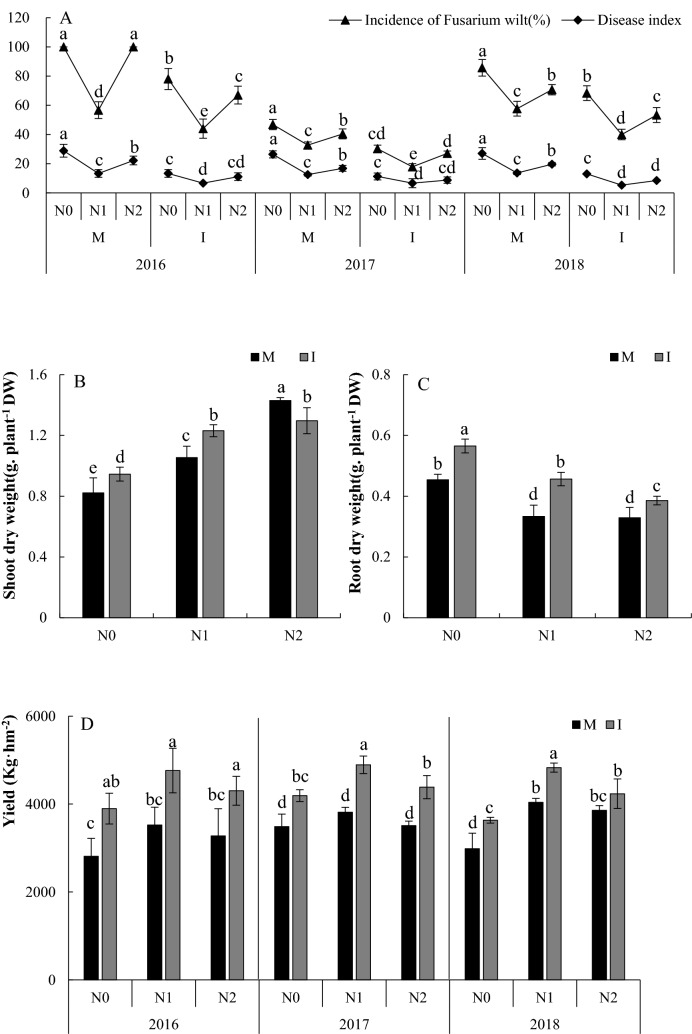
(**A**) Incidence and disease index of Fusarium wilt; (**B**) Faba bean shoot dry weight; (**C**) Faba bean root dry weight. (**D**) Yield of faba bean. All the values are presented as the mean ± SE. The different letters on the mean values of the same day indicate significant differences among the treatments (*P* < 0.05).

We measured the incidence and disease index of Fusarium wilt over a three-year period and found that the data of three years almost exactly followed the same trend (Fig. 3). An examination of the amounts of N applied indicate that the incidence and disease index of Fusarium wilt were reduced by these applications, and the N1 treatment was the lowest in both monocropping and intercropping conditions. An examination of the planting pattern indicated that intercropping reduced the incidence and disease index of Fusarium wilt and achieved significant effects under almost three different N conditions. In addition, we found the lowest incidence and disease index in 2017. Application with N significantly increased the shoot dry weight, and N2 treatment under monocropping was the highest. Compared with monocropping, intercropping significantly increased the shoot dry weight under the conditions of N0 and N1 and significantly reduced the shoot dry weight under the conditions of N2 (Fig. 3B). Applications of N reduced the root dry weight. Intercropping significantly increased the root dry weight compared with monocropping (Fig. 3C).

As shown in Fig. 3D, we found that, in 2016, application with N significantly increased the yield of faba bean under monocropping, and compared with monocropping, intercropping increased the yield of faba bean at three N levels, 38.54%, 35.16% and 31.41%, respectively. In 2017, application with N had no significant effect on the yield of faba bean. However, compared with monocropping, intercropping significantly increased the yield of faba bean, which was 20.19%, 28.26% and 24.89% higher, respectively. In 2018, application with N significantly increased the yield of faba bean under monocropping. Similarly, compared with monocropping, intercropping increased the yield of faba bean at three levels of N, 21.69%, 19.53% and 9.7%. Variance analysis of yield under different planting patterns and N level are shown in Table1.

**Table 1:** Variance analysis of yield under different planting patterns and N level.

Factor	*F*
A	4.479*
B	114.445**
C	28.330**
A × B	3.231^ns^
A × C	2.683^ns^
B × C	0.956^ns^
A × B × C	0.235^ns^

A: Year; B: Planting pattern; C: N level. * P < 0.05; ** P < 0.01; ns: Not significant.

## Discussion

Faba bean is very efficient nitrogen fixating crop, although we traditionally think that legumes do not need nitrogen application because they can fix nitrogen by Rhizobium, this is not the case. Some studies have proved that appropriate nitrogen fertilizer can help legumes nodulate and increase their nitrogen fixation^21^. In addition, Xiao and Guo et al. found that nitrogen application could significantly improve the yield of broad bean^22,23^. In this experiment, application with N and intercropping changed the components and quantity of organic acids exuded from the faba bean roots, among which the contents of tartaric acid and malic acid were the highest and regulated most significantly by application with N and intercropping. Therefore, we added tartaric acid and malic acid to observe their effect on the growth of FOF. The effect of allelochemicals depends on their concentration. We observed that tartaric acid inhibited the parameters of FOF, and the inhibition increased with the increase in concentration of tartaric acid. Malic acid also showed a similar trend, but the rate of inhibition of malic acid on spore germination of FOF was 100% at all concentrations. The application of N and intercropping with wheat promoted the exudation of tartaric acid and malic acid from faba bean roots, which were the highest under N1 intercropping conditions. The accumulation of tartaric acid and malic acid in the rhizosphere significantly inhibited the growth and reproduction of FOF, reduced the density of FOF in the rhizosphere, reduced the risk of infection of faba bean plants, and significantly controlled the disease, which may be an important mechanism for N application and intercropping to control Fusarium wilt of faba bean. The content of citric acid was the highest under conditions of N0 monocropping, and the combination of the application of N and intercropping tended to reduce the amount of citric acid exuded from faba bean roots. Some studies have shown that the amount of citric acid exuded from roots of plants will increase when mineral nutrition is deficient, which may be related to the ability of citric acid to activate nutrients^24^. In this study, when N was deficient, the roots exuded citric acid to activate the nutrients to alleviate nutrient stress. With the increase in input of N, the amount of citric acid returned to its normal level. The amount of fumaric acid exuded from faba bean roots decreased with the application of N and intercropping and was the lowest under N1 intercropping. A study by Li et al. found that fumaric acid can promote the chemotaxis of tobacco bacterial wilt, the formation of its cellular membrane, and its infection on tobacco^25^. In this study, an appropriate supply of N and a reasonable planting mode significantly reduced the amount of fumaric acid exuded from faba bean roots, which may reduce the chemotaxis of FOF to faba bean roots. This reduction could directly lower number of rhizosphere FOF of faba bean and the damage of FOF to faba bean, which may also be a mechanism to control Fusarium wilt. The amount and distribution of succinic and T-aconitic acids exuded from faba bean roots are very small and irregular. The role of succinic and T-aconitic acids in controlling Fusarium wilt of faba bean merits further study. As is well known, nitrogen (N) is an important factor that affects crop growth, development and limits crop yield in agricultural production, and it is the nutritional basis to ensure crop quality and yield. A balanced application of N is important for optimal disease resistance, because the effect of disease reduction is the most effective only when the N most suitable for plant growth is applied^26^. Within a reasonable range, with an increase in the input of N, the crops will have stronger resistance to disease, and the yield will also increase^27,28^. In this study, under N0 treatment, the faba bean was in a state of nutritional deficiency, and its various tissues and organs may not have been fully developed; its own defense system was incomplete; its disease resistance was poor, and pathogens were more likely to invade the plant, resulting in a high incidence and disease index and a low yield of faba bean. N is an essential element for plants to resist and recover from diseases^29^. With the increase in the input of N, under the N1 treatment, faba bean became more resistant to disease following this nutritional treatment. The organic acids exuded from faba bean roots also simultaneously reached their normal level, which contributed to the reduction in amount of FOF in the rhizosphere, increased faba beans resistance to Fusarium wilt and significantly reduced the damage caused by Fusarium wilt, finally significantly increasing the yield of faba bean. When the N input is continually increased, resulting in an excessive amount of N, we discovered that the N2 treatment increases incidence and disease index of faba bean and decreases its yield. This is probably owing to the toxicity of too much N to plants and its negative impact on the rhizosphere soil environment. In addition, there is evidence that an additional supply of N will increase the degree of interactions between plants and pathogens, because when N is not limited, pathogens are also more likely to obtain it^29^. For example, Haase et al. found that the supply of N would increase the amount of sugar and amino acids exuded from the roots of legumes, and sugar and amino acids could provide C and N sources for FOF and promote the amount of it in the rhizosphere, resulting in an increase in the risk of Fusarium wilt infection in faba bean^30^. Alternatively, during field conditions, an oversupply of elemental N would lead to excessive growth in the aboveground part of plants (Fig. 3) and closed canopy, leading to the deterioration of microclimate and breeding and transmission of other airborne pathogens, such as *Botrytis fabae,* the causal agent of chocolate spot disease on faba bean^3^. The combined damage of Fusarium wilt and air borne disease may be the important reason for reduction of faba bean yield under conditions of high N.

Intercropping, as the most effective way to increase species diversity, has long been used to control diseases. In this study, intercropping reduced the incidence and disease index of faba bean and increased its yield. The main reason was that intercropping regulated the composition and quantity of organic acids exuded from roots of faba bean, i.e., it can promote more “beneficial” organic acids, such as tartaric acid and malic acid, to be released from faba bean and reduce the exudation of “harmful” organic acids, such as fumaric acid, that inhibit the growth, propagation and activity of FOF and reduce its quantity in faba bean rhizosphere and the occurrence of diseases. In addition, intercropping with wheat significantly increased the dry weight of faba bean roots, which may indicate that the activity of faba bean root system increased, and then promoted the absorption of nutrients by faba bean root system, contributing to disease control and an increase in yield.

In conclusion, the application of N and intercropping can control Fusarium wilt of faba bean, which is closely related to the components and quantity of organic acids that exude from the faba bean roots. The contents of tartaric and malic acids were the highest and most important components because they can inhibit the growth and reproduction of FOF and contribute to the control of Fusarium wilt. This study shows that 90 kg ha^−1^ of N applied combined with wheat intercropping could significantly control Fusarium wilt and increase the yield of grain of faba bean. Based on these findings, increasing the supply of N will not only reduce the effect of controlling Fusarium wilt, resulting in yield loss but also cause environmental problems. Therefore, we do not recommend such a high input of N. We believe that 90 kg ha^−1^ of N combined with intercropping is the most effective way to control Fusarium wilt and should be incorporated into agricultural management practices.

## Materials and methods

### Materials

Local varieties of wheat (*Triticum aestivum* L. var. Yunmai 42) and faba bean (*Vicia faba* L. var. 92–24) were used as the plant materials. The seeds were purchased from Yunnan Academy of Agricultural Sciences, Kunming, China. FOF was isolated from an infected faba bean plot. Spore suspensions of the pathogen were obtained by adding 10 mL of sterile water to a Petri dish and rubbing the surface with a sterile L-shaped spreader. The suspension was then filtered through four layers of cheesecloth. The spore concentration was determined using a hemocytometer^31^.

### Design of the hydroponic experiment

**Figure 4:**
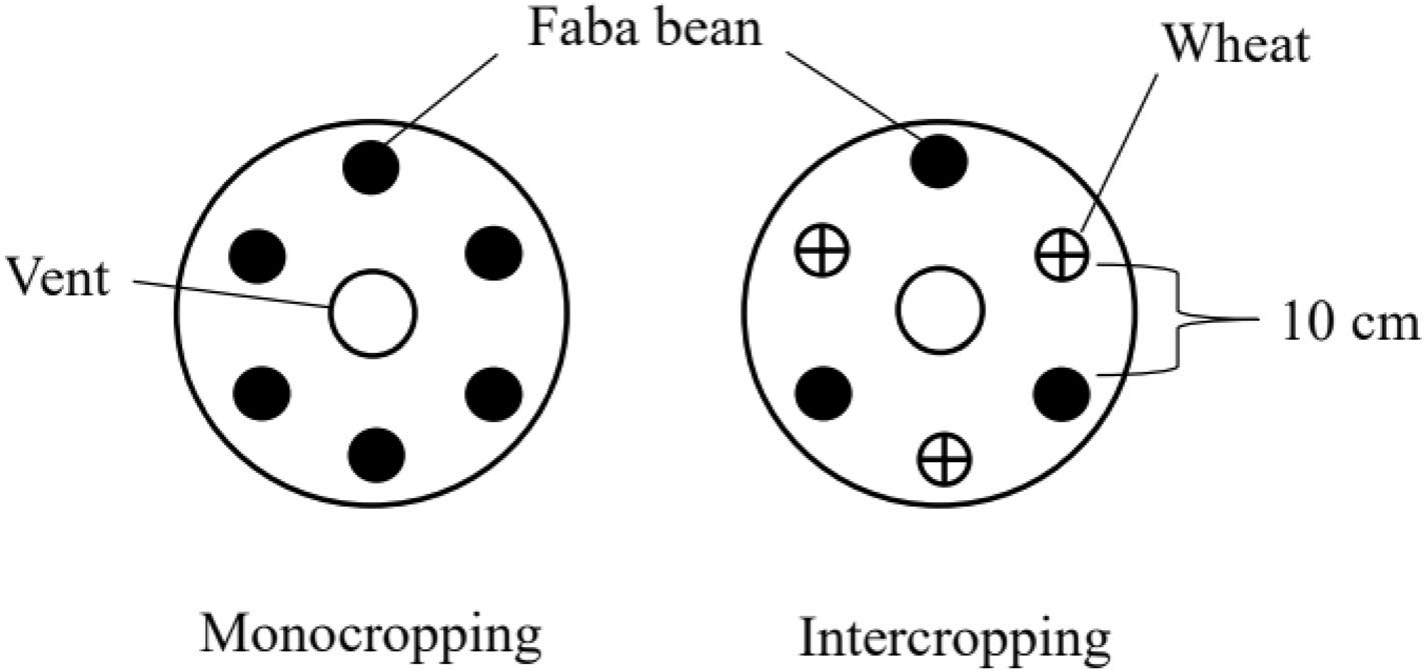
Diagram of the planting patterns in the greenhouse.

The hydroponic experiment was conducted from September to December 2017 in the glasshouse of Yunnan Agricultural University. The illumination was 14 h a day/10 h of night, and the temperature was 26 °C/22 °C. A two-factor randomized block design was used in the experiment. Factor A was the N application level, and three treatments with four replicates were established: no nitrogen (N0), conventional nitrogen (N 1:2 mmol/L Ca[NO_3_]_2_) and high nitrogen (N 2:5 mmol/L Ca[NO_3_]_2_). Factor B was the planting pattern: faba bean monocropping, which included six faba bean seedlings grown alone in pot culture; wheat/faba bean, which included three faba bean seedlings grown, and nine wheat seedlings grown on the other side; the distance between the faba bean seedlings and the wheat seedlings was 10 cm with four replicates per treatment (Fig. 4). The nutrient solution formulation used was (mmol/L): K_2_SO_4_ 0.75, MgSO_4_ 0.65, KCl 0.1, KH_2_PO_4_ 0.25, H_3_BO_4_ 0.001, MnSO_4_ 0.001, CuSO_4_ 0.0001, ZnSO_4_ 0.001, (NH_4_)_6_Mo_7_O_24_ 0.000005, Fe-EDTA 0.2^32^. Calcium ions in the form of CaCl_2_ (2 mmol/L) were added to the no-N treatment.

Seeds of the same size and plumpness with a complete seed coat were selected, soaked in 10% (v/v) H_2_O_2_ for 30 min, cleaned with deionized water, and then soaked in saturated CaSO_4_ for 12 h until the seeds were full and bloated. The seeds were then wrapped with wet filter paper, immersed in a saturated CaSO_4_ solution, germinated in a culture chamber at 25 °C, and the cotyledons were expanded and cultured in 1/2 nutrient solution for 24 h before being cultured in a total nutrient solution. The nutrient solution was circulated throughout the whole cultivation process using a ventilation pump. The volume of nutrient solution was 4 L, and it was stored in a plastic pot (28 cm in the upper diameter, 20 in the lower diameter and 17 cm high), and the nutrient solution was changed every three days. The pH of the nutrient solution was maintained between 6.20 and 6.30. A completely random arrangement was used in this experiment.

### Collection and determination of root exudates

After 40 days of transplantation, the faba bean seedlings were harvested by sampling. The faba bean roots were cleaned with tap water to remove impurities, followed by cleaning with deionized water. Three faba bean seedlings were immersed in a plastic cup containing 300 mL deionized water in each pot. Two to three drops of concentrated phosphoric acid were added to inhibit microbial activity. The root exudates were collected after culture under light for four hours (10:00–14:00 per day). The collected root exudates were filtered through a 0.45 micron Millipore membrane (Merck-Millipore, Darmstadt, Germany), freeze-dried and stored at − 20 °C. During collection, each cup with faba beans was covered with black plastic to block pollution and light^33,34^. The lyophilized powder of the root exudates was dissolved in deionized water and stored for analysis^35^.

The faba bean exudates were filtered through a 0.22 um filter, and the contents of organic acids were determined. The organic acids were isolated and identified from faba bean root exudates using high performance liquid chromatography (HPLC) (Agilent 1200, Agilent Technologies, Waldbronn, Germany). All the chemicals purchased were analytical reagent quality, and the solvents used were HPLC spectral grade. The reagents were purchased from Sigma-Aldrich (St. Louis, MO, USA). The major peaks were identified by comparing the retention times with those of matching standards. The organic acids were determined using a chromatographic column, Zorbax SB-Aq (4.6 mm × 250 mm, 5 mm) (Agilent Technologies) as described by Lv et al.^33^. The detector wavelength was 215 nm, and the column temperature was 35 °C. The standard organic acids for the HPLC analysis included oxalic, malic, citric, succinic, T-aconitic and fumaric acids.

### Effect of organic acids on the mycelial growth of FOF

Organic acids were added to the PDA media to prepare the new media with the CK (sterile water control), C1 (50 mg L^−1^), C2 (100 mg L^−1^), C3 (200 mg L^−1^), C4 (400 mg L^−1^), C5 (800 mg L^−1^) and C6 (1,600 mg L^−1^) concentrations. The final volume of culture medium for each plate was 20 mL. The same age of the FOF mycelia with the same growth and thickness of the culture medium was obtained using a 0.8 mm diameter punch and inoculated into the prepared plate center. The colony diameter was measured at 28 °C for six days, and four replicates were used per treatment.

$$ {\text{Growth}}\;{\text{impact}}\;{\text{index}}\;\left( \% \right) = \left( {{\text{colony}}\;{\text{diameter}}\;{\text{of}}\;{\text{treated}} - {\text{colony}}\;{\text{diameter}}\;{\text{ of}}\;{\text{ control}}} \right)/\left( {{\text{colony}}\;{\text{diameter}}\;{\text{of}}\;{\text{control}} - 0.{8}} \right) \times {1}00 $$

### Effect of organic acids on FOF sporulation

Bilay’s medium was mixed with an organic acid of the concentration to be measured and evenly mixed^36^. The FOF spore suspension prepared in a 0.1 mL aliquot was inoculated in each treatment (approximately 2 × 10^5^ CFU mL^−1^). The culture was shaken at 28 °C (120 rpm) for three days, diluted and then coated on PDA medium. After two days of culture at 28 °C, the number of colonies was recorded. This number was used to calculate the concentration of spores that was present in the liquid culture of Bilay’s medium after three days of growth^36^. There were four replicates per treatment.

$$ {\text{Sporulation}}\;{\text{ impact}}\;{\text{index}} = \left( {{\text{number}}\;{\text{of}}\;{\text{spores}}\;{\text{ in}}\;{\text{a}}\;{\text{treatment}} - {\text{control}}} \right)/{\text{control}} \times {1}00 $$

### Effect of organic acids on FOF spore germination

FOF colonies cultured on PDA plates for seven days were washed with aseptic water, and the spores were collected by filtration through four layers of gauze and diluted into spore suspensions with a concentration of less than 1 × 10^3^ CFU mL^−1^. Organic acids were added to prepare a plate with the CK (sterile water control), C1 (50 mg L^−1^), C2 (100 mg L^−1^), C3 (200 mg L^−1^), C4 (400 mg L^−1^), C5 (800 mg L^−1^) and C6 (1,600 mg L^−1^) concentrations in in 2% (w/v) water agar medium. Finally, the culture medium volume of each plate was 20 mL. Each plate was coated with a 0.1 mL spore suspension, and the number of colonies on the plate was recorded two days after culture at 28 °C. There were four replicates per treatment.

$$ {\text{Germination}}\;{\text{impact}}\;{\text{index}}\;\left( \% \right) = \left( {{\text{number}}\;{\text{ of}}\;{\text{colonies}}\;{\text{treated}} - {\text{control}}} \right)/{\text{control}} \times {1}00 $$

### Field test design

**Figure 5:**
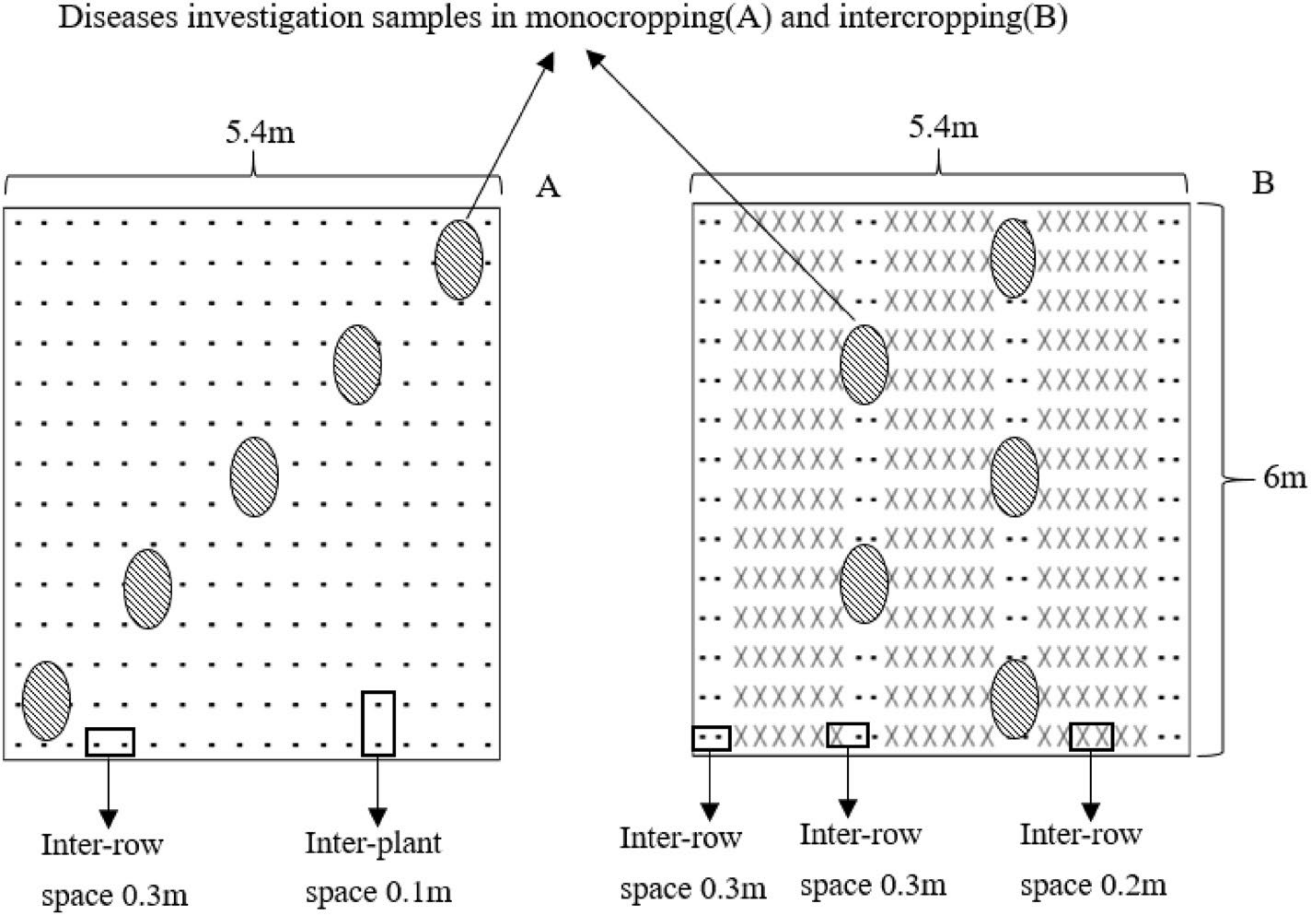
Diagram of the planting patterns in the field experiments: (**A**) the monocropping faba bean plot, (**B**) the intercropping faba bean with wheat plot (− represents faba bean and × represents wheat).

Field experiments were conducted in Lubiao Town (24°61′ N, 102°18′ E), Anning City, China, from October 2015 to June 2018. The monthly average temperature and rainfall during the field experiments are shown in Fig. 5. Paddy soil was used, and the soil is classified as a Haplic Lixisol (FAO-UNESCO, 1988). The physicochemical properties of the soil were as follows: total organic carbon = 23.2 g kg^−1^; total N = 1.90 g kg^−1^; alkali hydrolyzed N = 119.0 mg kg^−1^; available P = 56.5 mg kg^−1^; available K = 123.4 mg kg^−1^ and pH 6.4 (1:2, soil: water ratio). In the field experiment, two factors were established. Factor A was the N level, and there were three levels: no N application (N0: wheat and faba bean, no N application), conventional N application (N1: faba bean, 90 kg ha^−1^; wheat, 180 kg ha^−1^) and high N application (N2: faba bean, 135 kg ha^−1^; wheat, 270 kg ha^−1^). Each treatment was repeated four times. Factor B was the planting pattern: faba bean monocropping (M) and wheat/faba bean intercropping (I). Random fissure plots were used in the field. There were four replicates per treatment and 24 plots in total.

The fertilizers tested included urea (including N 46%), common superphosphate (calculated by P_2_O_5_) and potassium sulfate (calculated by K_2_O). The P fertilizer was 112.5 kg ha^−1^; the K fertilizer was 112.5 kg ha^−1^, and no organic fertilizer was applied. The fertilizers were all used as base fertilizer and applied at one time.

**Figure 6:**
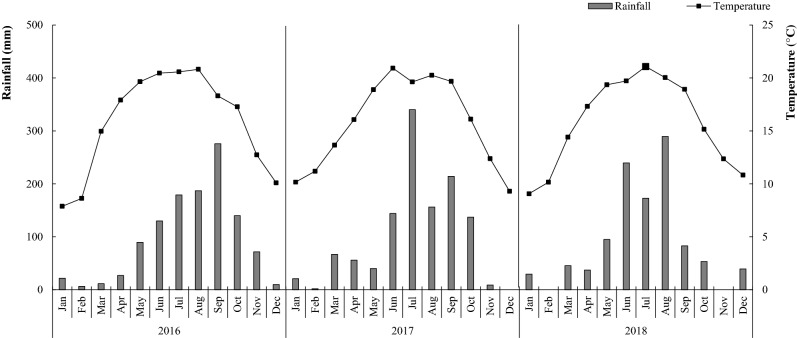
The monthly average temperature and rainfall during the field experiments.

The area of the plots was 5.4 × 6 m = 32.4 m^2^. Detailed information about the planting patterns and methods of disease investigation are shown in Fig. 6. One-meter wide faba bean strips were planted all around the experimental field as protective rows.

The faba bean and wheat were sown approximately October 21 each year, and the harvest date was approximately April 23 of the next year. No pesticides, fungicides and herbicides were applied throughout the growth period. Other management was carried out according to the local agronomic customs.

### Assessment of disease incidence

Diseases were investigated at the peak of disease (flowering stages) of faba bean. Five markers were selected according to the diagonal method in the monocropping plots. Three plants were surveyed for each marker, and 15 faba bean plants were surveyed for each plot. In the intercropping plot, five markers were selected on two faba bean bands (the first with two markers, and the second with three markers). Three plants were investigated with each marker, and 15 faba bean plants were investigated in each plot. The classification standard of Fusarium wilt of faba bean was as follows: 0 indicated no visible symptoms; 1 indicated mild wilt symptoms on the leaf and stem; 2 indicated moderate wilt symptoms; 3 indicated severe wilt symptoms, and 4 indicated plant death. The disease incidence was defined as the percentage of the number of infected plants to the number of investigated plants. The disease index (DI) was calculated as:

$$\mathrm{Disease} \; \text{index}=\frac{\Sigma (\mathrm{Number} \; \text{of} \; \text{diseased} \; \text{plants} \; \text{at} \; \text{each} \; \text{level}\times \mathrm{ level})}{\mathrm{The} \; \text{highest} \; \text{level } \times \mathrm{total} \; \text{number} \; \text{of} \; \text{plants} \; \text{investigated}}\times 100$$

### Plant dry biomass analysis

Sampling was conducted at the same time of the disease investigation, i.e., the faba bean plants after disease investigation were taken as the sampling plants. The faba bean plants were dried in an oven for 15 min at 105 °C and then placed at 70 °C for three days. The dry weight of each plant was measured with an electric balance.

### Assessment of yields

Faba beans were manually reaped when they were fully mature. The grain yield and 100-grain weight of the faba bean were determined, and they were converted into hectare yield.

### Statistical analysis

Statistical analyses were performed using SPSS software v. 13.0 (SPSS, Inc., Chicago, IL, USA). All the data were expressed as the means ± standard errors. Significant differences between the treatments were evaluated using a 2-factor analysis of variance (ANOVA), followed by Tukey’s test at the 5% probability level.

### Ethics statement

All methods were carried out in accordance with relevant guidelines and regulations.

## Abbreviations

FOF: *Fusarium oxysporum* F. sp. *fabae*
N: Nitrogen
M: Monocropping
I: Wheat/faba bean intercropping
